# Impact of Antimicrobial Stewardship on Antimicrobial Utilization and Resistance Patterns in a Tertiary Care Hospital in Western Maharashtra

**DOI:** 10.7759/cureus.80012

**Published:** 2025-03-04

**Authors:** Neelam R Attar, Sara S Dhanawade, Divya Yadav, Jalandhar Nikam

**Affiliations:** 1 Microbiology, Bharati Vidyapeeth (Deemed to be University) Medical College & Hospital, Sangli, IND; 2 Paediatrics and Child Health, Bharati Vidyapeeth (Deemed to be University) Medical College & Hospital, Sangli, IND; 3 Quality Assurance, Bharati Vidyapeeth (Deemed to be University) Medical College & Hospital, Sangli, IND

**Keywords:** antibiotic policy, antimicrobial resistance, antimicrobial stewardship, antimicrobial utilization, aware classification, ceftriaxone, culture-based therapy, daily defined doses, irrational fixed-dose drug combination, surgical prophylaxis

## Abstract

Background: Non-judicious use of antibiotics by health professionals has been identified as an area for interventions and improvement by the World Health Organization for controlling antimicrobial resistance. Following the Indian Council for Medical Research (ICMR) guidelines, we established an antimicrobial stewardship (AMS) program at the 950-bedded multispecialty private sector hospital located in western Maharashtra by the end of the year 2021.

Aim and objective: The purpose of this study was to evaluate the impact of an AMS program intervention on the utilization of antibiotics and resistance patterns of organisms isolated from the patients.

Result: A significant reduction in the utilization of ceftriaxone (23.3 to 6.75), piperacillin-tazobactam (7.7 to 6.0), amikacin (9.03 to 5.15), clindamycin (6.25 to 5.75), linezolid (5.8 to 4.8), and ceftazidime (0.9 to 0.2) in defined daily doses (DDD/100 bed days) was seen after intervention. Antibiotic resistance decreased in gentamicin, amikacin, and teicoplanin. Overall antibiotic consumption reduced from 1,681.0 to 1,420.0 DDD/100 days. Culture-based therapy increased from 61% to 90%. Surgical prophylaxis compliance increased from 58% to 96%.

Conclusion: The constant perseverance of the AMS team of our hospital had a positive impact on reducing the overall consumption of antibiotics. Stringent infection prevention and control practices, timely provision of treatment guidelines, frequent interactions and discussions with treating doctors, audits by clinical pharmacists, and feedback to the doctors along with various training programs and sensitization sessions by the AMS team have brought significant behavioral changes among the treating physicians.

## Introduction

The global pandemic of antimicrobial resistance (AMR) has impacted every region of the world, jeopardizing the effectiveness of infection prevention and treatment. Almost 1.27 million deaths were attributed, and five million were associated with AMR in 2019, and now by 2050, the death toll is estimated to reach 10 million annually [1].

As per the World Health Organization (WHO) Bacterial Pathogen Priority List of 2024, carbapenem-resistant Enterobacterales, carbapenem-resistant *Acinetobacter*, cephalosporin-resistant Enterobacterales, and rifampicin-resistant *Mycobacterium tuberculosis* have been identified as the critical pathogens. The list highlights the impact of these pathogens in terms of burden, as well as issues related to transmissibility, treatability, and prevention options. It also guides stakeholders in research and development and investing in AMR [2].

India carries the largest burden of antibiotic-resistant organisms and is, hence, referred to as the AMR capital of the world [3]. Rising population, malnutrition, poverty, lack of access to healthcare, and lack of access to diagnostic facilities have all added to the ever-increasing problems of infectious diseases in the country. Drug resistance has been exacerbated by self-medication with broad-range antibiotics, easy access to over-the-counter medications, and medical professionals' careless use of antibiotics.

In 2015, WHO adopted the Global Action Plan for Antimicrobial Resistance (GAP-AMR) in collaboration with the Food and Agriculture Organization and the World Organisation for Animal Health. The plan provided five objectives for the member states to tackle the development of AMR. Phase 1 and Phase 2 of the National Action Plan were introduced by the Indian government in 2017 and 2022, respectively. In 2018, the Indian Council of Medical Research (ICMR) formulated the antimicrobial stewardship (AMS) program (AMSP) guidelines consisting of the main components of the application, which will reduce the effects of AMR [4]. Accreditation bodies collaborated by establishing an AMSP as a crucial component of the fundamental purpose. Following the guidelines, we established an AMSP at the 950-bedded multispecialty private sector hospital located in western Maharashtra by the end of the year 2021. An AMSP has been advocated as the most essential tool to combat AMR. It involves sensitizing healthcare staff, providing treatment guidelines, monitoring antibiotic prescription practices, and discouraging irrational antibiotic use through feedback. The purpose of this study was to evaluate the impact of AMSP intervention on the utilization of antibiotics and the resistance pattern of organisms isolated from the patients.

## Materials and methods

A prospective analytical study was conducted on antimicrobial utilization and AMR from the year 2019 to 2023. The study included all patient care areas of a 950-bed tertiary care hospital. Our hospital consists of six intensive care units, seven surgical wards, four medicine and pediatrics wards, and four high-dependency units. As the interventions and recommendations were evidence-based and regarded as the standard of care, the Institutional Ethics Committee of Bharati Vidyapeeth (Deemed to be University) Medical College and Hospital, Sangli, approved this study (553/24) and waived the requirement for informed consent. Data was gathered both prior to and following the intervention (2019-2021).

CDC recommends seven core components in the implementation of AMSP [5]. They consist of hospital leadership commitment, where the hospital provides necessary human, financial, and information technology resources. Physicians and pharmacists will be accountable for program management and outcomes. The clinical pharmacist will lead implementation efforts to improve antibiotic use and apply infection-based, provider-based, pharmacy-based, microbiology-based, and nursing-based interventions. The impact of interventions will be tracked by monitoring antibiotic prescribing practices, the prevalence of *Clostridioides difficile* infection, and resistance patterns among hospital pathogens. Antibiotic prescribers and nurses will be provided with regular updates on antibiotic use and resistance. They will be educated on appropriate antibiotic-prescribing practices.

Based on these elements, our process of AMSP implementation began with the formation of an AMS committee by the end of the year 2021. It consisted of consultants from various departments, an infection control officer, a microbiologist, a pharmacologist, and five clinical pharmacists. The goals of the program were to promote the safe, effective, and rational use of antibiotics, slow down AMR by optimizing antibiotic use, and reduce overall healthcare costs.

In addition to the national recommendations, the AMS team and experts used the most recent antibiogram pattern to update the antibiotic policy. The clinical pharmacist, along with the microbiologist, conducted daily rounds in patient care areas. The first step was to look for what antibiotics were used for empirical therapy and whether it was in compliance with the antibiotic policy of the hospital. As preauthorization was not feasible with our setup, an antibiotic justification form was attached to the patient's file, and residents were asked to fill out forms within 24 hours of starting antibiotics. Forms were checked daily by microbiologists and clinical pharmacists during rounds. Consultants and residents were sensitized to using culture-based therapy. Antibiotic modifications (escalation or de-escalation) based on the culture report were monitored. Reminders for reviewing antibiotic continuation were given to consultants on day seven or 10. Consultants and residents were invited for a weekly meeting to discuss cases where antibiotic use was not justified. Additional details, including demographics, diagnosis, history of prior hospitalization and treatment, whether the infection was community- or hospital-acquired, and whether a gram-positive or gram-negative infection was suspected, were provided by the antibiotic justification form. Every quarter, AMSP meetings were held to discuss data pertaining to several AMS metrics. We began with readily attainable quality metrics like policy adherence indicators (adherence to policy, culture-based therapy, antibiotic choice, and timing for surgical prophylaxis), administrative compliance indicators (correct dose and duration of antibiotics), and antibiotic utilization indicators (defined daily doses (DDD)).

All consultants were given access to the quarterly AMS indicator data for improvement and feedback. Consultants, resident doctors, and nursing staff were sensitized frequently throughout the year through guest lectures, quizzes, and poster competitions. Antibiotic policy was provided in booklet form to each resident and consultant for easy access. A WhatsApp (Meta Platforms, Inc., Menlo Park, CA, US) group was created where the AMS team communicated several criteria for optimal antibiotic usage in order to improve communication between the team and consultants and residents. Infection control practices were simultaneously implemented. Through active surveillance and healthcare personnel training, appropriate steps were made to prevent hospital-acquired infections.

An antibiogram of six groups of organisms frequently isolated from the patient sample was included in the study. *Escherichia coli*, *Klebsiella* spp., *Acinetobacter* spp., *Pseudomonas* spp., *Staphylococcus* spp., and *Enterococcus* spp. were monitored for five years for resistance patterns. The following isolates were excluded from the study: (a) isolate from surveillance and (b) repeat isolate from the same sample.

Conventional methods were used to process the patient's sample, and VITEK 2 (bioMerieux, Marcy-l'Étoile, France) was used to identify the organism. Minimum inhibitory concentration (MIC) results were interpreted using Clinical Laboratory Standards Institute guidelines [6]. Antibiogram patterns of each of these isolates were analyzed at the end of the year. The antibiogram pattern was stratified depending on the type of isolates, source, and area and circulated as an annexure in the antibiotic policy for the consultant’s information.

To calculate antibiotic consumption, we retrospectively collected data from medical records and pharmacy software data. The Anatomical Therapeutic Chemical (ATC)/DDD is a universal parameter suggested by WHO for calculating the antibiotic utilization of every antibiotic and combination [7]. The usage of antibiotics was calculated by using the ATC/DDD per 100 bed days. The prescribed antibiotics were also classified based on the Access, Watch, and Reserve (AWaRe) classification. Additional data consisting of the use of irrational drug combinations was also extracted. The antibiotics that fall under the ATC classification but were dispensed to the outpatient and treatment after discharge were excluded from the study. Microsoft Excel 2021 edition (Microsoft Corp., Redmond, WA, US) was used to organize and clean the imported and calculated data.

After five years of monitoring the use of common antibiotics (piperacillin-tazobactam, ceftriaxone, ciprofloxacin, imipenem, gentamicin, teicoplanin, amikacin, clindamycin, linezolid, meropenem, vancomycin, and ceftazidime), the patterns of resistance were compared to the use of these antibiotics. Statistical analysis was done using Microsoft 365 (Microsoft Corp., Redmond, WA, US) and IBM SPSS - 29 (IBM Corp., Armonk, NY, US). Descriptive statistics and Spearman rank correlation were applied.

## Results

**Figure 1:**
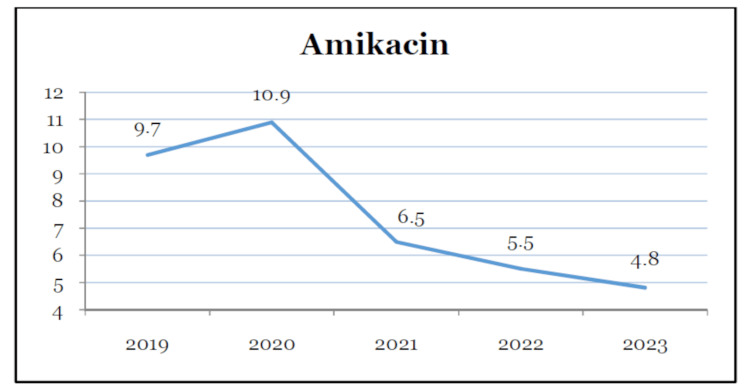
Decreasing trend of amikacin utilization over the five-year period

**Figure 2:**
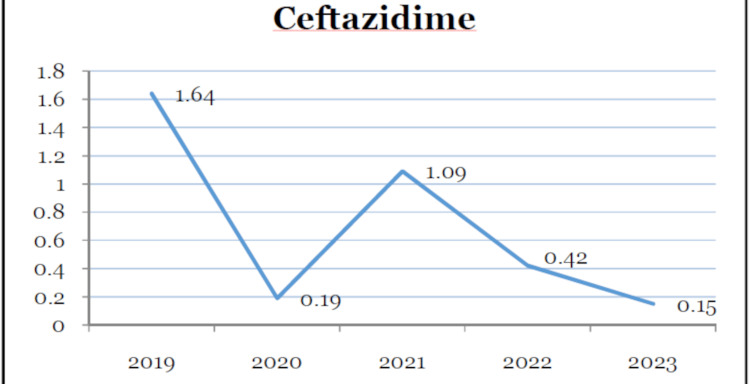
Decreasing trend of ceftazidime utilization (DDD/100 bed days) over the five-year period DDD: defined daily dose

Table 1 provides antibiotic utilization in DDD/100 bed days over a period of five years. The overall consumption of all antimicrobials differed throughout the study period. There was a gradual reduction in the utilization of amikacin and ceftazidime (Figures 1, 2). Ceftriaxone was the highest consumed antibiotic for three consecutive years before AMSP intervention. The utilization reduced drastically after the year 2022. Piperacillin-tazobactam was also the highest consumed drug in the year 2020. The usage reduced afterward. The utilization reduced in the years 2022 and 2023.

**Table 1:** Antibiotic utilization over the period of five years in DDD/100 bed days DDD: defined daily dose

Drugs	2019	2020	2021	2022	2023
Piperacillin-tazobactam	5.1	13.2	4.8	5.6	6.53
Ceftriaxone	13.4	27.7	29	3.4	10.1
Ciprofloxacin	0.45	0.41	0.31	0.32	0.39
Imipenem	0.1	0.09	0.01	0.08	0.08
Gentamicin	2.19	1.5	1.3	2.5	2.8
Teicoplanin	0.15	0.08	0.24	0.7	0.9
Amikacin	9.7	10.9	6.5	5.5	4.8
Clindamycin	3.94	6.74	8.05	5.4	6.1
Linezolid	4.9	6.4	6.3	4.3	5.3
Meropenem	3.2	4.4	3.8	3.4	5.5
Vancomycin	0.93	0.26	0.87	1.2	0.95
Ceftazidime	1.64	0.19	1.09	0.42	0.15

We analyzed the resistance pattern of *E. coli*, *Klebsiella*, *Acinetobacter* spp., *Pseudomonas* spp., *Staphylococcus* spp., and *Enterococcus* over a period of five years from 2019 to 2023 (Table 2). Among all the isolates analyzed over five years, *E. coli* (29%) was the most frequently isolated organism every year, followed by *Klebsiella* spp. (22%), *Staphylococcus* (16%), *Acinetobacter* (12%), *Pseudomonas* (11%), and *Enterococcus* spp. (8%). *E. coli* showed a reducing resistance for ceftriaxone from 92% to 89% in the years 2022 and 2023. Imipenem, meropenem, amikacin, and gentamicin resistance in *E. coli* decreased during the AMSP intervention in the years 2022 and 2023. Resistance of *Klebsiella *to ceftriaxone, piperacillin-tazobactam, and carbapenem has remained the same in all five years. Resistance to gentamicin has increased since 2021. There is a significant dip in the amikacin resistance pattern during the years 2021 and 2022 (from 56% to 24%). *Acinetobacter* shows an initial decrease in resistance to meropenem, imipenem, gentamicin, and amikacin for the year 2021, followed by an increase in resistance for the years 2022 and 2023. Resistance to piperacillin-tazobactam has remained almost the same for five years. There is no significant change in the resistance pattern of *Pseudomonas* to imipenem, meropenem, gentamicin, and amikacin. Ceftazidime and piperacillin-tazobactam showed a reduction in resistance in the years 2021 and 2022. Seventy percent of isolates were methicillin-resistant *Staphylococcus aureus* (MRSA) during all five years. Gentamicin and vancomycin initially showed high resistance in the year 2020, followed by dropping resistance in the following years. *Staphylococcus* has constantly shown high-level resistance to fluoroquinolones in all five years. Linezolid and clindamycin resistance has increased in 2021 and 2022 and dropped to 28% in 2023. *Enterococcus* has shown high resistance to ciprofloxacin in all years. Vancomycin resistance increased in the year 2022. Twenty-five percent of isolates of *Enterococcus* were vancomycin-resistant (VRE) in all five years.

**Table 2:** Antimicrobial resistance pattern over a period of five years

	Drugs	2019	2020	2021	2022	2023
Escherichia coli	Piperacillin-tazobactam	30	57	52	51	52
	Imipenem	22	51	56	36	33
	Meropenem	2	42	55	35	34
	Gentamicin	48	44	58	38	40
	Amikacin	18	49	45	21	20
	Ceftriaxone	79	81	85	92	89
*Klebsiella* spp.	Piperacillin-tazobactam	64	70	68	67	72
	Imipenem	41	41	61	64	64
	Meropenem	55	55	59	61	68
	Gentamicin	38	38	58	57	57
	Amikacin	56	56	6	3	24
	Ceftriaxone	79	79	85	85	86
*Acinetobacter* spp.	Piperacillin-tazobactam	76	85	92	89	92
	Imipenem	63	79	56	80	85
	Meropenem	38	87	55	79	88
	Gentamicin	81	92	72	76	82
	Amikacin	66	84	65	74	84
	Ceftriaxone	89	96	91	79	86
*Pseudomonas* spp.	Piperacillin-tazobactam	47	67	50	11	28
	Imipenem	54	67	54	60	55
	Meropenem	53	68	50	64	56
	Gentamicin	42	43	50	59	50
	Amikacin	55	71	67	65	51
	Ceftazidime	36	64	32	14	30
*Staphylococcus* spp.	Gentamicin	45	63	51	40	31
	Clindamycin	20	27	40	70	26
	Teicoplanin	10	9	6	5	4
	Vancomycin	2	18	11	9	8
	Ciprofloxacin	69	83	79	87	86
	Linezolid	30	28	48	55	28
*Enterococcus* spp.	Teicoplanin				10	17
	Vancomycin	3	35	30	42	23
	Ciprofloxacin	70	89	74	81	87
	Linezolid				8	13

Spearman's correlation was used to identify the correlation between antibiotic utilization and resistance among the isolates (Table 3). Spearman’s rank correlation is used to check whether the utilization of antibiotics and resistance are correlated. The correlation value R greater than or equal to 0.8 is a strong correlation. A minus sign indicates a negative correlation, and no sign is a positive correlation.

**Table 3:** Spearman’s correlation between antibiotic utilization and resistance A p-value lesser than 0.05 is significant NS: not significant; S: significant

Organism	Drug	Spearman's correlation R	Significance	Result
Escherichia coli	Piperacillin-tazobactam	0.5642	0.32172	NS
	Imipenem	-0.6668	0.2188	NS
	Gentamicin	-0.8	0.10409	NS
	Amikacin	0.4	0.50463	NS
	Meropenem	0.3	0.62384	NS
	Ceftriaxone	-0.6	0.2847	NS
Klebsiella	Piperacillin-tazobactam	0.6	0.2847	NS
	Imipenem	-0.64889	0.23615	NS
	Meropenem	0.4616	0.4377	NS
	Gentamicin	-0.1054	0.866	NS
	Amikacin	0.6668	0.2188	NS
	Ceftriaxone	0.9	0.0373	S
Acinetobacter	Piperacillin-tazobactam	-0.1539	0.8048	NS
	Imipenem	0.0513	0.9347	NS
	Meropenem	0.9	0.03739	S
	Gentamicin	0.3	0.62384	NS
	Amikacin	-0.1026	0.8696	NS
	Ceftriaxone	0.9	0.03739	S
Pseudomonas	Piperacillin-tazobactam	-0.1	0.87289	NS
	Imipenem	-0.1539	0.7998	NS
	Meropenem	-0.3	0.6284	NS
	Gentamicin	-0.3077	0.6143	NS
	Amikacin	-0.7	0.18812	NS
	Ceftazidime	-0.2	0.74706	NS

There is a high positive and significant correlation for ceftriaxone in *Klebsiella* and *Acinetobacter* (R = 0.9, p = 0.03739). It indicates that an increase in utilization increases resistance. This also stands true for meropenem in *Acinetobacter*.

**Figure 3:**
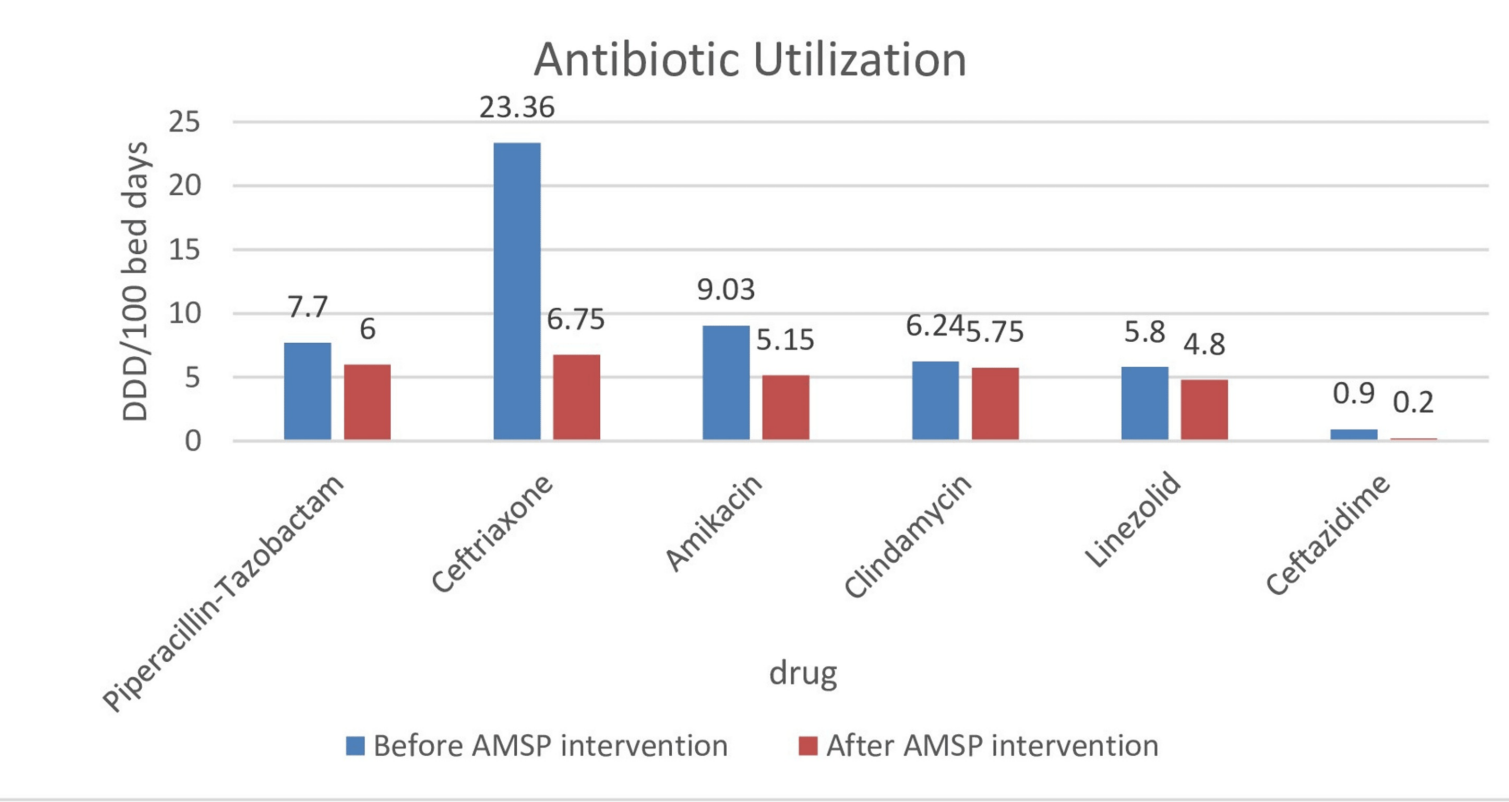
Utilization of antibiotics before and after intervention (defined daily doses (DDD)/100 bed days) AMSP: antimicrobial stewardship program

A significant reduction in the utilization after AMSP intervention was observed in ceftriaxone, piperacillin-tazobactam, amikacin, clindamycin, linezolid, and ceftazidime (Table 4 and Figure 3). Antibiotic resistance decreased in gentamicin, amikacin, and teicoplanin following AMSP intervention (Table 5).

**Table 4:** Impact of antimicrobial stewardship program (AMSP) interventions on utilization

Drugs	Before AMSP intervention	After AMSP intervention
Piperacillin-tazobactam	7.7	6
Ceftriaxone	23.36	6.75
Ciprofloxacin	0.39	0.35
Imipenem	0.06	0.08
Gentamicin	1.66	2.65
Teicoplanin	0.15	0.8
Amikacin	9.03	5.15
Clindamycin	6.24	5.75
Linezolid	5.8	4.8
Meropenem	3.8	4.45
Vancomycin	0.68	1
Ceftazidime	0.9	0.2

**Table 5:** Impact of antimicrobial stewardship program interventions on antimicrobial resistance

Drugs	Before intervention	After intervention
Piperacillin-tazobactam	61.0%	73.0%
Ceftriaxone	84.8%	86.3%
Ciprofloxacin IV	77.3%	86.0%
Imipenem	49.7%	56.0%
Gentamicin	53.1%	45.0%
Teicoplanin	8.3%	4.5%
Amikacin	46.0%	38.8%
Clindamycin	29.0%	38.0%
Linezolid	35.3%	41.5%
Meropenem	48.3%	56.0%
Vancomycin	16.8%	20.8%
Ceftazidime	56.0%	78.0%

As an additional finding, AMS quality indicators were also monitored simultaneously during interventions. Policy compliance reached 96% after interventions. There is remarkable improvement in the administration of the correct dose and duration of antibiotics. The practice of culture-based treatment increased from 61% to 90%. Overall antibiotic consumption, which was 1,681.0 DDD/100 bed days before intervention, dropped by 15%. As irrational drug combination usage was also discouraged, a reduction of 28% was also observed (Table 6).

**Table 6:** Antimicrobial stewardship achievements DDD: defined daily dose

Antimicrobial stewardship quality indicators	Before	After
Policy adherence indicators		
Empirical antibiotics were as per policy	73%	96%
Surgical prophylaxis was as per policy	58%	96%
Administrative compliance indicators		
Antibiotic was administered in the correct dose and duration	83%	93%
Culture-based antibiotic therapy used	61%	90%
Surgical antibiotic prophylaxis is given in the correct time frame	79%	98%
Antimicrobial usage outcome indicator		
Overall antibiotic consumption	1,681.0 DDD/100 bed days	1,420.0 DDD/100 bed days
Irrational fixed-dose combination usage	98.1 (9.3%)	70.3 (4.8%)

Antibiotic utilization as per the AWaRe classification was analyzed (Table 7). Watch group antibiotics form the major component of the antibiotic class used. Access group antibiotic utilization ranged between 30% and 28% during all five years. 

**Table 7:** Antibiotic utilization trend as per the AWaRe classification AWaRe: Access, Watch, and Reserve

AWaRe class	2019	2020	2021	2022	2023
Access	519.8 (30.9%)	275.9 (21.6%)	475.2 (22.8%)	213.9 (16.7%)	437.1 (28.0%)
Watch	1,078.8 (64.2%)	938.4 (73.3%)	1,437.7 (69.1%)	1,007.4 (78.7%)	1,027.8 (65.9%)
Reserve	82.7 (4.9%)	65.3 (5.1%)	169.1 (8.1%)	59.2 (4.6%)	94.9 (6.1%)

## Discussion

AMS has long been identified as an essential tool to ensure appropriate antibiotic usage among healthcare personnel and the general population. Rather than using restrictive measures as suggested in studies [8], we intended to bring behavioral changes through regular communication with the consultants on a case-by-case basis. Senior consultants from medicine and pediatrics provided counseling for antibiotic modifications. Feedback was also provided through structured feedback forms. These actions increased the postgraduate students' confidence in their ability to use antibiotics appropriately. Over time, postgraduate students and consultants began to rely on clinical pharmacists and microbiologists to recommend antibiotics. In several antibiotics, loading doses that were overlooked were also fixed. The majority of studies on antibiotic consumption conducted so far have been point prevalence studies. We were able to assess the long-term effects of AMSP interventions on physicians' behavior thanks to our five-year study.

India ranked first in antibiotic consumption from 2000 to 2010 [9]. Broad-spectrum antibiotics like fluoroquinolones, cephalosporins, and macrolides were frequently used for both inpatient and outpatient cases. Ceftriaxone was the most abused cephalosporin in our hospital while being the most affordable and accessible medication. Ceftriaxone was given to patients with viral diseases such as dengue, malaria, and IC bleeds due to a lack of knowledge and unidentified fear. Ceftriaxone misuse was also observed in a study by Jabeen et al. [10]. We were able to cut its usage by about 71%, from 23.36 DDD/100 bed days to 6.75 DDD/100 bed days. For surgical prophylaxis, surgeons typically used amikacin in combination with cephalosporin. Since amikacin was a tuberculosis (TB) reserve medication, we discouraged its use and saw a 42% reduction. During the two critical years of the COVID-19 epidemic, piperacillin-tazobactam was widely provided as advised by the Ministry of Health. By the end of 2021, it was a routine practice to treat each and every respiratory tract infection with piperacillin-tazobactam. Our hospital provided the physicians with different diagnostic modalities, which could help them differentiate viral cases of pneumonia (polymerase chain reaction (PCR) respiratory panels). These measures not only reassured the physicians but also contributed to a decrease in the use of piperacillin-tazobactam. Overall consumption of all antibiotics dropped by 15% after two years of AMSP intervention.

With the reduced use of ceftriaxone over the five-year period, a reduction in resistance among *E. coli* isolates was seen. There was no change in ceftriaxone resistance in *Klebsiella*, though *Klebsiella* spp. showed decreased resistance to amikacin following reduced consumption. Our hospital, being a tertiary care center, receives antibiotic-exposed and referred patients for further management. This amplified the burden of multidrug-resistant organisms (MDROs) such as *Acinetobacter*, *Pseudomonas*, and MRSA. There was no significant change in the resistance pattern of *Acinetobacter*, *Pseudomonas*, and *S. aureus* over the five-year period. When the average pattern of resistance to gentamicin, teicoplanin, and amikacin was compared before and after the intervention, it showed a big drop.

As evident from other studies [11-13], we could find a similar positive correlation between antibiotic utilization and resistance. The correlation was statistically significant only for ceftriaxone use in *Klebsiella *and *Acinetobacter* and meropenem in *Acinetobacter*. Studies by Laxminarayan and Chaudhury [14] have suggested multifactorial causes in the development of AMR, consumption being only one of the factors.

To evaluate the effects of our two years of interventions, we looked at a number of indicators. The parameters included loading dose, maintenance dose, frequency, duration of therapy, de-escalation and escalation, rational fixed-dose combination (FDC) usage, culture-based therapy, policy adherence for empirical therapy, treatment change based on AMS team suggestion, IV to oral switch, and medication interactions.

The areas where aggressive intervention was still needed were de-escalations of antibiotic class. The compliance for de-escalation was better with the surgical department. Patients were given IV antibiotics for two to three days and then shifted down to oral antibiotics as per policy recommendations. We could not achieve significant de-escalation in critical care units, the reason being the majority of patients were previously treated with higher antibiotics already. They were colonized or infected with MDRO strains. This led to prolonged treatment with high-end antibiotics and an inability to de-escalate.

We achieved a significant improvement in the administration of the loading dose (93% compliance) as against the results seen in the study by Singh et al. [15], where compliance was very low (38%) for meropenem and polymyxin B. Loading doses for drugs like polymyxin B, colistin, tigecycline, and vancomycin were added in the policy for reference. The use of colistin, polymyxin B, and tigecycline started in the year 2022. These drugs were utilized as last-resort measures to treat carbapenem-resistant organisms. We initiated sensitization through interactions with treating physicians during daily rounds and provided them with literature for reference. Corrections were suggested in both loading and maintenance doses wherever required.

The use of dual antibiotics with an overlapping spectrum and dual-anaerobic cover was a frequent observation before intervention. The practice was more prevalent in surgical departments. Interventions by the AMS team did result in behavioral changes among the prescribing consultants, leading to a reduction in use.

According to a study by Koya et al. [16], a huge number of antibiotics are not permitted by central drug regulators, and a considerable portion of the Indian population takes FDC from formulations outside the National List of Essential Medicines (NLEM). We have reduced the usage of illogical FDCs in our hospital by sensitizing prescribers and routinely revising the hospital drug formulary in accordance with the NLEM list.

Culture-based therapy was not a standard practice in the hospital before AMSP. Only 60% of patients were treated using culture-based antibiotics. There was excellent compliance among pediatricians to use targeted therapy and modify empirical choice based on culture reports. Compliance was extremely low among medicine, surgery, orthopedics, and ENT. This scenario was more or less the same in other hospitals around the globe, as evident from various studies [17,18]. The AMS team at our hospital trained the residents and consultants on diagnostic stewardship. Residents were encouraged to assess the need to send samples during daily rounds. Residents were provided with an MIC guiding table for antibiotic selection and VITEK AST report interpretation. We increased the practice of targeted therapy from 60% to 90% postintervention.

Swamy et al. [19] employed a patient risk stratification model to identify patients at risk of having MDRO pathogens. The majority of patients in his study belonged to risk groups 2 and 3 that were already exposed to antibiotics. This justified the use of higher antibiotics and also reduced the chances of group 1 patients receiving higher antibiotics. Instead of using the risk stratification model to identify patients with MDRO, we screened previously hospitalized patients for bloodstream infections, urinary tract infections if catheterized, and respiratory tract infections if ventilated on day one of admission. Patients who were only colonized and not infected with MDRO were isolated, closely monitored, and treated as per policy for antibiotic-naïve patients. Those with true infections received higher antibiotics as per antibiotic susceptibility reports.

WHO introduced the AWaRe classification in the year 2017 to support AMS efforts at local, national, and global levels. WHO's 13th General Program of Work 2019-2023 recommended that countries should have 60% of total antibiotic consumption from the Access group of antibiotics, and the ratio of Access to Watch should be close to 1.5 [20]. The ratio seen in our study throughout the five years was 0.4, 0.2, 0.3, 0.2, and 0.4, respectively, which was far from the recommendation. There were similar observations seen in other studies conducted in India [21,22]. With a high prevalence of extended-spectrum beta-lactamases (ESBLs), AmpC co-producers, carbapenem-resistant Enterobacterales, and *Acinetobacter* in our hospital, the Watch group of antibiotics remained the only option to treat patients. The issue of resistant organisms in India is evident from various studies conducted so far [23]. Both ICMR and the National Centre for Disease Control (NCDC) have reported alarming levels of resistance among *E. coli* and *Klebsiella* for third-generation cephalosporins, fluoroquinolones, carbapenems, and beta-lactam/beta-lactamase inhibitor combinations in the 2022 annual report [24,25].

As indicated by Sahani et al.'s [26] findings in their study, there is a paucity of AMSP intervention studies that include an education component as an important measure to strengthen stewardship activities. In order to encourage students to engage, we placed a strong emphasis on regular trainings for undergraduates and offered AMSP and hospital infection control as elective postings. Hospital infection control, diagnostic stewardship, sample collection and transportation techniques, and AMS were among the specific subjects covered in the introduction training given to postgraduate students.

Limitations of the study

As we used three years of AMSP preintervention data and compared it with two years of postintervention, we had to ensure uniformity in the antibiotics studied throughout the period. Antibiotics like colistin, tigecycline, and polymyxin B were neither tested nor used till the year 2022. Hence, we could not include the consumption of these antibiotics in our study. Issues like antimicrobial cycling, dose optimization, and cost calculations were not addressed in this study. Area-wise consumption of antibiotics from the critical care unit and wards was not looked into in the present study. Clinical outcome indicators (morbidity and mortality) also were not addressed.

## Conclusions

The constant perseverance of the AMS team of our hospital had a positive impact on reducing the overall consumption of antibiotics by 15%. We brought down the utilization of ceftriaxone by 71%, amikacin by 42%, piperacillin-tazobactam by 22%, clindamycin by 8%, and linezolid by 17%. There was a decrease in resistance seen in *E. coli* for ceftriaxone and *Klebsiella* for amikacin. There was a positive correlation seen between the utilization of ceftriaxone and resistance among *Klebsiella* and *Acinetobacter*. A similar correlation was also seen in *Acinetobacter* for meropenem. Antibiotic policy adherence improved by 31%, surgical prophylaxis compliance by 65%, and culture-based therapy by 47%. The use of irrational FDC was reduced by 22%.

Rational use of antibiotics in humans, animals, and farming plays a major role in slowing down the ever-evolving AMR. While the government takes regulatory action at all other levels, it is the ethical responsibility of the treating physicians to use antibiotics judiciously. Stringent infection prevention and control practices, timely provision of treatment guidelines, frequent interactions and discussions with treating doctors, audits by clinical pharmacists, and feedback to the doctors along with various training programs and sensitization sessions by the AMS team have brought significant behavioral changes among the treating physicians.
